# Effects of Shuganjianpihuatanxingqi decoction on mild subclinical hypothyroidism: A SPIRIT compliant randomized controlled trial study protocol

**DOI:** 10.1097/MD.0000000000013183

**Published:** 2018-11-09

**Authors:** Litao Bai, Jing Zhao, Jialiang Gao, Fei Li, Fan Wei, Jun Li, Yao Xiao, Xu Han, Yaoli Wang, Danwei Wang, Fangying Wu, Junping Wei

**Affiliations:** a Department of Endocrinology, Guang’anmen Hospital; b China Academy of Chinese Medical Sciences; c Department of Cardiology, Guang’anmen Hospital, China Academy of Chinese Medical Sciences; d Department of Endocrinology, Beijing University of Chinese Medicine; e Department of Endocrinology, Shuguang Hospital Affiliated to Shanghai University of Traditional Chinese Medicine Shanghai; f Department of Endocrinology, Beijing Changping District Integrated Traditional Chinese Medicine and Western Medicine Hospital Beijing, People's Republic of China.

**Keywords:** mild subclinical hypothyroidism, protocol, randomized controlled trial, traditional Chinese medicine

## Abstract

**Introduction:**

Mild subclinical hypothyroidism (SCH) can cause depression, fatigue, cognitive dysfunction, or other hypothyroid symptoms, and even progress to hypothyroidism. The treatment of mild SCH is controversial. Shuganjianpihuatanxingqi decoction (SD) is a frequently prescribed Chinese herbal medicine in patients with mild SCH. However, scientific evidence is needed to confirm the therapeutic effect of SD.

**Methods and analysis:**

This study is a randomized, double-blind, and controlled clinical trial. A total of 228 participants with the diagnosis of mild SCH will be randomly assigned to the SD or placebo group in a ratio of 1:1. Participants will receive treatment for 12 weeks and undergo 12-month follow-up. The primary outcome measure is the thyroid-stimulating hormone level, and secondary outcomes will be the differences in the results of Thyroid-related Quality of Life Questionnaire, blood lipids, and Traditional Chinese Medicine Symptom Score Scale between baseline and at 12 weeks after intervention.

**Ethics and dissemination:**

The study has been approved by Guang’anmen Hospital of China Academy of Chinese Medical Sciences (no.2018-005-ky-01). The trial results will be published via peer-reviewed journals and the Clinical Research Information Service.

**Trial registration number:**

ChiCTR1800015781 (approval date: 20 April 2018).

## 1 Introduction

Hypothyroidism is a common disease of thyroid hormone deficiency. In adults, the most common symptoms of hypothyroidism include fatigue, lethargy, constipation, cold intolerance, weight gain, sound changes, and dry skin. In severe cases, the absence of treatment can result in mortality.^[1]^ Subclinical hypothyroidism (SCH) is a condition of elevated serum thyroid stimulating hormone (TSH) with normal serum free thyroxine (fT4).^[2]^ Studies have indicated a high prevalence rate of SCH in the United States, ranging from 3% to 18% in the adult population, with higher rates among women and the elderly.^[3,4]^ Another study showed that the prevalence rate of untreated SCH in Spain was 4.6%.^[5]^ A cross-sectional study of 10 cities in China reported that the number of patients with SCH increased from 3.22% to 16.7% in the past decade.^[6]^ Patients with SCH are more prone to depressive symptoms, decreased quality of life, cognitive function, and memory loss than those with normal thyroid function.^[7–9]^ In addition, thyroid hormones have a variety of complex effects on the heart, blood vessels, bone, and brain.^[10]^ Patients with SCH show a higher risk of hyperlipidemia, atherosclerosis, hypothyroidism symptoms, and cardiovascular events.^[11,12]^ The treatment of SCH remains controversial.^[13]^ In general, a thyrotropin cutoff level of 10 mIU/L is used to distinguish between mild and more severe SCH.^[14,15]^ While most experts recommend levothyroxine therapy in patients with SCH with TSH level of greater than 10 mIU/L, there is lack of consensus on whether patients with mild SCH with TSH levels less than 10 mIU/L should be treated.^[16]^ A Cochrane systematic review showed that levothyroxine replacement therapy in patients with SCH did not improve survival or reduce cardiovascular morbidity. Data on health-related quality of life and symptoms showed no significant differences between the intervention groups.^[17]^ Excessive levothyroxine intervention may cause SCH.^[16]^ Therefore, most patients with mild SCH are recommended for follow-up observation. However, patients often complain of depression, fatigue, cognitive function, or other hypothyroid symptoms. In addition, under increasing health awareness among patients, mild SCH that has not been treated tends to aggravate the patients’ anxiety and depression. As a result, many patients seek Chinese herbal medicine for treatment; in addition, attempts have been made to provide Chinese medicine treatment in patients with mild SCH.

Chinese medicine has a long history of treatment in patients with thyroid disease. Over a period of thousands of years, Chinese medicine has accumulated rich experience and numerous drugs to target thyroid disease. In the third century BC, TCM doctors recorded some typical symptoms of thyroid dysfunction and named the disease Ying disease. In the Sui dynasty, a Chinese medicine book, the *Zhu Bing Yuan Hou Lun* indicated that thyroid disease is related to regional factors and the patients’ mental state. In the Tang Dynasty, iodine-containing herbal Chinese medicine was used to treat patients with iodine-deficient goiter. Currently, traditional Chinese medicine (TCM) is widely used to treat patients with thyroid diseases in China. Some preliminary studies reported the therapeutic effects of TCM in patients with thyroid diseases. A clinical study reported that Chinese medicine can reduce the size of goiter in patients with multinodular and diffuse goiter.^[18]^ A randomized trial reported that a Chinese herbal dispersion formula improved the treatment outcomes of antithyroid drug treatment in patients with hyperthyroidism with neurologic manifestations of Graves’ disease by means of modulating the levels of IL-2, IL-8, and IL-17.^[19]^*Prunella vulgaris L.* promoted apoptosis of well-differentiated human thyroid carcinoma cells.^[20]^ Prunellae oral liquid can be used as an adjunct treatment in patients with goiter.^[21]^ A case report revealed potential effectiveness of Anemarrhena Bunge decoction in patients with Graves’ disease with resistance to meilizazole.^[22]^ In the treatment of hypothyroidism, Chinese herbal medicine has been used alone or in combination with levothyroxine therapy to relieve the symptoms of patients’ discomfort.^[23,24]^

SD, a formula comprised of multiple herbs, such as, *Pinellia ternata (Thunb.) Breit.*, *Magnolia officinalis Rehd. et Wils* and *Prunella vulgaris L*., and so on is a frequently prescribed TCM in patients with mild SCH. Based on TCM theory, it can improve the symptoms of depression, fatigue, and hypothyroidism. SD evolved on the basis of the Banxia-houpu decoction. *Jin Gui Yao Lue* by the reputed TCM doctor Zhang Zhongjing of the Han Dynasty stated that a Banxia-houpu decoction could be used to improve depression and throat discomfort. An in vivo study reported that Banxia-houpu decoction can increase the anti-stress effect through the hypothalamus-pituitary-adrenal gland axis and modification of stress behaviors.^[25]^ Water extract of *Magnolia officinalis Rehd. et Wils* has antidepressant-like effects by improving the hypothalamus-pituitary-adrenal axis function, promoting expression of brain-derived neurotrophic factor in the hippocampus, and increasing hippocampal neurogenesis.^[26]^ Autoimmune thyroiditis is the most common endogenous cause of SCH.^[27]^ The active ingredients of *Magnolia officinalis Rehd. et Wils* can strengthen innate immune signaling responses and modulate inflammation^[28,29]^; whereas, the active ingredients of *Poria cocos (Schw.) Wolf* can regulate mammalian immune cells, with potential for use in treating Th2-mediated immune disorders.^[30]^

In a preliminary study including a small sample, SD showed effect to improve symptoms in patients with mild SCH and reduce TSH levels; however, there have been a few high-quality trials and more evidence is needed to prove the therapeutic effect of SD. This study aims to evaluate the effectiveness and safety of SD in patients with mild SCH using strict high-quality methodology per Consolidated Standards of Reporting Trails (CONSORT) statement for randomized controlled trials of herbal medicine.^[31]^

The results of this trial will hopefully provide clinical evidence for the treatment of patients with mild SCH.

## 2 Methods

### 2.1 Objectives

The aim of this trial is to assess the efficacy and safety of SD in patients with mild SCH.

### 2.2 Study design

**Figure 1:**
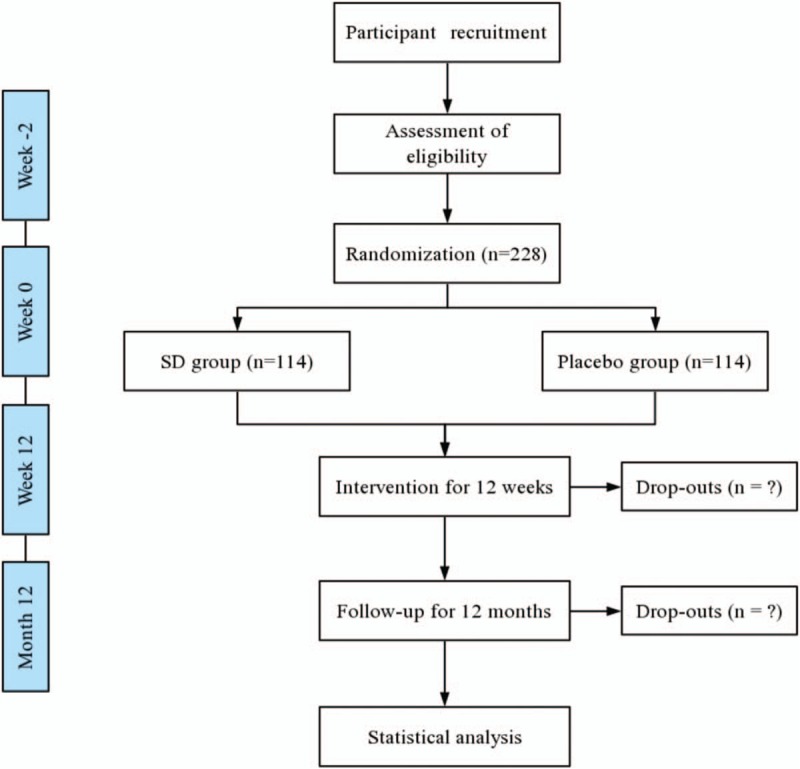
Flow diagram. SD = Shuganjianpihuatanxingqi decoction.

This is a double-blind, randomized, and controlled trial to investigate the efficacy and safety of SD compared with that of the placebo. The study will be conducted in 3 centers in China: Guang’anmen Hospital of China Academy of Chinese Medical Sciences, Xiyuan Hospital of China Academy of Chinese Medical Sciences, and Wangjing Hospital of China Academy of Chinese Medical Sciences. Patients with mild SCH will undergo a standardized baseline evaluation before treatment including detailed history taking, physical examination, and laboratory testing. Included participants will be randomly divided into two groups, an SD group and the other that will receive placebo. The efficacy and safety of SD will be assessed after 12 weeks’ treatment and 12 months’ follow-up at drug withdrawal. All visits will be recorded in Care Report Forms (CRFs). The flow-chart is shown in Figure 1, and the time-point of assessment is shown in Table 1. The development of the protocol of this study is per Standard Protocol Items: Recommendations for Interventional Trials (SPIRIT) guideline.^[32]^

**Table 1:**
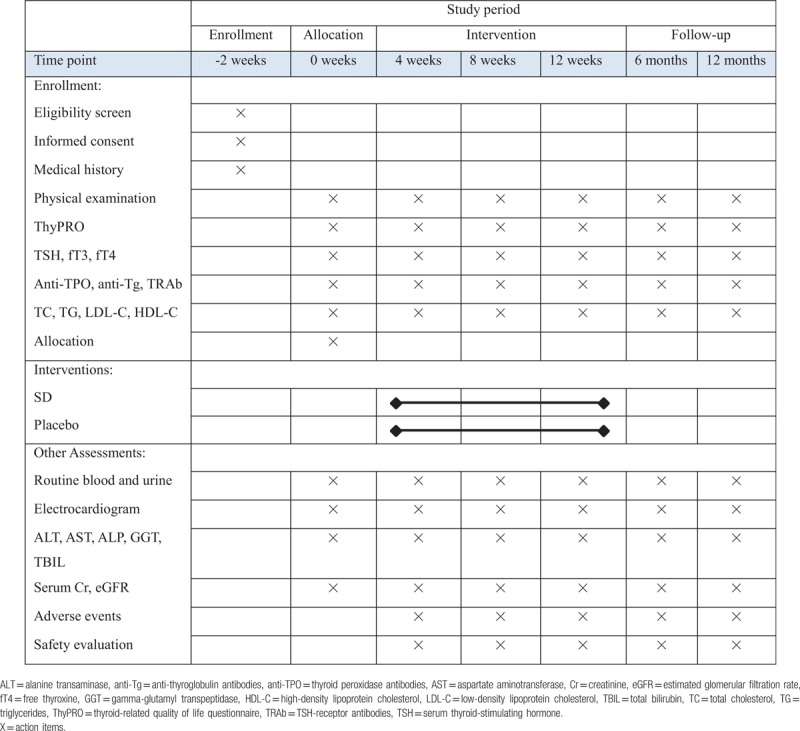
Schedule of enrollment, intervention, and assessment.

### 2.3 Study setting and recruitment

Between December 2018 and December 2020, a total of 228 patients will be recruited in the three centers mentioned above through posters, hospitals’ website, and networks. The research assistants will manage the recruitment, and the endocrinologists will diagnose the participants.

### 2.4 Participants

#### 2.4.1 Diagnosis criteria

The diagnostic criteria for mild SCH are based on the criteria set by the American Association of Clinical Endocrinologists and the American Thyroid Association in 2012.^[33]^

#### 2.4.2 Inclusion criteria

1. Diagnosis based on persistently elevated TSH levels (4.6–10.0 mU/L), with fT4 within the reference range.
2. Age of minimum of 18 years to a maximum of 70 years.
3. Capability of complete compliance and completion of follow-up.
4. Willingness to provide written informed consent.

#### 2.4.3 Exclusion criteria

1. Current prescription of levothyroxine, antithyroid drugs, amiodarone, or lithium.
2. Recent thyroid surgery or radioiodine therapy within 1-year of the study's start date.
3. History or presence of clinically relevant cardiovascular, renal, metabolic, hematological, neurological, psychiatric, systemic, or infectious disease or malignant tumor.
4. Severe mental disorder.
5. Recent hospitalization for major illness or elective surgery within 1-year of the study's start date.
6. Pregnancy or lactation, intention to become pregnant, and/or not using appropriate contraceptive methods.
7. Allergy to components of the agents used in the study.
8. Terminal illness.
9. Genetic problems such as galactose intolerance, Lapp lactose deficiency, or glucose-galactose malabsorption.
10. History of alcoholism or substance abuse.
11. Recent participation in other clinical trials.

### 2.5 Randomization, allocation concealment, and blinding

Participants will be randomly assigned to either the SD or placebo group in a 1:1 ratio. Randomization using Statistics Analysis System (SAS) software will be performed by independent staff statisticians. The randomization numbers are kept in opaque sealed envelopes.

Allocation concealment will be performed by the Data Management Center of Guang’anmen Hospital. Randomization code generation and drug blinding will be implemented independent of the data collection, evaluation, and analysis. The drug administrator at each participating medical center will enroll patients sequentially on the basis of screening order. Both participants and investigators will be blinded to the allocation until completion of the trial. To achieve blinding, the same kind of packaging will be used for both the drug and placebo in each group. The placebo will be matched to the corresponding medicine in terms of size, color, shape, taste, and smell by adding artificial pigment.

Twenty-four-hour emergency code break and medical information will be provided by the Data Management Center of Guang’anmen Hospital. Each subject will be allowed access to the investigator through telephone in case of emergency. In case of a severe adverse event, an administrator will unblind the patient information as an emergency and provide relevant treatment.

### 2.6 Intervention

All investigators will be clinical doctors with certification in endocrinology with at least 3 years’ experience and will receive standardized training for the diagnostic interview before the start of the research. Two clinicians will diagnose the mild subclinical hypothyroidism. Participants in the intervention group will take SD orally twice a day for 12 weeks, whereas participants in the control group will take placebo orally twice a day for 12 weeks. Patient visits will be required at 0, 4, 8, and 12 weeks.

The placebo is composed of 95% starch, 5% SD, and a very small amount of bitterant. The SD and placebo (Sichuan New Green Co., Ltd., Chengdu, Sichuan, China) will be produced at a dosage of 4 g, with shelf-life of 2 years.

### 2.7 Follow-up

All included participants will be re-evaluated at 6, and 12 months through phone calls or as outpatients. Participants with worsened thyroid function will receive a supply of relevant medicine and a written withdrawal schedule.

### 2.8 Outcome measures

#### 2.8.1 Primary outcomes

The primary endpoint is the change in Thyroid-related Quality of Life Questionnaire (ThyPRO) results from baseline to week 12, with severity rating on a scale of 0 to 100 at 0, 4, 8, and 12 weeks from the start of the treatment period.^[10,34]^

#### 2.8.2 Secondary outcomes

The secondary endpoint is the change in TSH from baseline to week 12. Biochemical measurements of TSH levels will be performed in the laboratory of each center at 4 weeks’ interval. In addition, the change in blood lipids, including the total cholesterol, triglyceride, high-density lipoprotein will be measured.

#### 2.8.3 Safety assessments

Safety will be assessed in terms of the vital signs, and routine renal function, liver function, blood, urine, stool, and electrocardiogram test results obtained during the screening period and after 12 weeks of treatment. Adverse events, vital signs, and laboratory examinations will be recorded on a case report form before and after patients take their medication at every visit. Adverse events are defined as any unexpected sign or symptom during the trial period, and participants will be asked to inform clinicians about the occurrence of any adverse events while taking their medication. All information about adverse events will be recorded in detail, such as the duration and extent of the adverse event, relationship with the trial medicine, and name of the suspected drug. Common adverse events may include gastrointestinal side effects, such as stomach discomfort, nausea, and diarrhea, which are not expected to be severe. However, if serious adverse events occur that may lead to death or require extended hospitalization, the participants will be asked to quit the clinical trial as soon as possible, and proper treatment will be provided.

### 2.9 Statistics

#### 2.9.1 Sample size

This study is a trial of a new therapeutic regimen of SD treatment. In a similar study conducted previously on selenium supplementation, the total sample size was 192 participants.^[35]^ Allowing for a 20% withdrawal rate, we plan to include 228 patients in this trial.

#### 2.9.2 Data analyses

Statistical Package for the Social Sciences (SPSS) version 17.0 (SPSS, Inc., Chicago, IL) software will be used for data analysis. An intention-to-treat analysis will be conducted for patients who underwent treatment at least once. Continuous data with normal distribution will be presented as mean ± standard deviation, and those without normal distribution as median (interquartile range). Statistical comparisons will be analyzed using the student *t* test, and Wilcoxon rank sum test for continuous data and *χ*^*2*^ test for categorical data. Differences between groups will be evaluated using the *χ*^*2*^ test or Wilcoxon rank sum test. All statistical tests will be 2-sided tests, and *P*-value of < .05 will be considered statistically significant.

#### 2.9.3 Data collection and management

All researchers involved in the study will be qualified physicians. Researchers will receive training in standard operating procedures for trial execution, evaluation of the TCM symptoms, biological sample collection, and handling. According to the original observation records, investigators at all centers will complete the CRFs in an accurate and timely manner. The administrators of the Guang’anmen Hospital of China Academy of Chinese Medical Sciences will visit each center regularly to confirm the quality of data collection and facilitate problem-solving. All documents will be properly classified, preserved under confidential conditions, and archived.

#### 2.9.4 Participant retention and withdrawal

Participants may withdraw from the trial at any time for any reason, and the reason will be recorded in the CRFs. The investigator will inform the participants that they have the right to withdraw from the research project and will be provided with standardized treatment to ensure their safety under the following conditions: rapidly decreasing estimated glomerular filtration rate to below 30 mL/min/1.73 m^2^, alanine transaminase or aspartate aminotransferase level 2-fold higher than the normal limits, or serum creatinine level beyond the normal range; serious complications or deterioration of existing health condition; poor participant compliance, such as actual drug use of less than 80% or more than 120% of the prescribed dose; or use of proscribed drugs during the study. Participants in this trial will be provided with cost-free drugs and scheduled examinations. Necessary examinations and treatments will also be provided for any adverse event.

#### 2.9.5 Ethics and dissemination

The trial will be conducted in accordance with the principles of the Declaration of Helsinki (2013 version). The trial design will follow the principles set out in the Good Clinical Practice guidelines for the appropriate clinical use of TCM. The trial is approved by the Ethics Committee of Guang’anmen Hospital of China Academy of Chinese Medical Sciences (approval no. 2018-005-ky-01). After the clinicians provide a complete explanation to the participants, written informed consent will be obtained from the participants before treatment intervention. The trial was registered at the Chinese Clinical Trial Registry (ChiCTR1800015781) on April 20, 2018. The trial will fully comply with the SPIRIT reporting guidelines.^[26]^ The trial will help to demonstrate if SD is effective and safe for patients with mild SCH and the results will be published in peer-reviewed journals to ensure widespread dissemination. All protocol amendments will be approved by the Institutional Review Board prior to execution.

## 3 Discussion

As a valuable medical treatment, TCM is beneficial to many people currently. The essence of TCM lies in treatment based on the differentiation of disease syndromes. Currently, many TCM doctors regard TCM as a basic or complementary therapy for patients with thyroid disease. SD may improve the symptoms of mild SCH and is usually prescribed for patients with mild SCH. This trial aims to examine the efficacy and safety of SD in comparison with placebo to treat mild SCH. The quality of randomized controlled trials of Chinese medicine has many problems.^[27]^ Issues mainly include faulty study design and methodology and lack of training of the investigators. To ensure the quality of this study and reach a reliable conclusion, the experimental design and study performance are under strict quality control. For the experimental design, we used the Consolidated Standards of Reporting Trials (CONSORT) Extension for Chinese Herbal Medicine Formulas.^[28]^ At each center, training sessions will be provided to explain the study protocol, implementation of the TCM syndrome pattern differentiation, and the standard operating procedures; an independent laboratory at each center will be used to manage the biochemical measurements. The findings of this trial may enable alternative treatment in patients with mild SCH; in addition, they may provide scientific evidence for the use of SD to relieve the patients’ symptoms and control TSH levels. The small sample size of this study is a limitation. Nevertheless, the results will provide new evidence for the effectiveness of SD treatment in patients with mild SCH.

## Acknowledgments

The authors thank the staff and patients participating in the study.

## Author contributions

**Conceptualization:** Litao Bai, Jing Zhao, Junping Wei.

**Data curation:** Jun Li.

**Formal analysis:** Danwei Wang.

**Methodology:** Jun Li, Yao Xiao, Yaoli Wang, Fangying Wu.

**Supervision:** Junping Wei.

**Writing – original draft:** Litao Bai, Jing Zhao.

**Writing – review & editing:** Jialiang Gao, Fei Li, Fan Wei, Xu Han, Junping Wei.
